# Review of Factors Related to the Thyroid Cancer Epidemic

**DOI:** 10.1155/2017/5308635

**Published:** 2017-05-02

**Authors:** Yihao Liu, Lei Su, Haipeng Xiao

**Affiliations:** Department of Endocrinology, The First Affiliated Hospital of Sun Yat-sen University, 58 Zhongshan Road 2, Guangzhou, China

## Abstract

Thyroid cancer is the most common endocrine cancer, of which the incidence has dramatically increased worldwide in the past few decades. The reasons for the observed rapid increase still are not fully understood, but evidence suggests that overdiagnosis, with the advancement in detection methods and screening policies, is not the sole driver of the substantial increase of the incidence. However, the effect of environmental/lifestyle factors remains speculative other than that of radiation exposure at a young age. This review tries to give a balanced view of debated factors leading to the thyroid cancer epidemic, to offer some alternatives in understanding the controversies, and to suggest potential directions in the search of modifiable risk factors to help reduce thyroid cancer.

## 1. Introduction

During the past several decades, the incidence of thyroid cancer worldwide has increased at a higher rate than any other cancer, while mortality rates for thyroid cancer have remained relatively stable [1–3]. Albeit a less lethal form of cancer than many other malignancies, the social and economic impact of the growing incidence of thyroid cancer is not trivial. In 2013 alone, the estimated overall societal cost of care for well-differentiated thyroid cancer of U.S. patients diagnosed after 1985 is $1.6 billion [4]. In China, the disability-adjusted life years (DALY) and years of life lost (YLL) due to premature mortality attributed to thyroid cancer were 133.5 thousand person-years and 114.2 thousand person-years, respectively, in 2013 [5]. Interestingly, the increase in thyroid cancer incidence was mainly driven by increase of cases of the papillary subtype, while the increase for follicular or medullary subtypes was to a much lesser extent [6]. This discrepancy could have been the result of improved detection of small cancers and indolent microcarcinomas (<1 cm), 87.4% of which were of the papillary subtype [1].

Whether the substantial increase of incidence of thyroid cancer is solely the result of improved detection (i.e., an apparent increase) or to some extent attributable to known or new risk factors causing a real change in the incidence (i.e., a true increase) is still up for debate [7–10]. Here, we offer an overview of the evidence lending support to either side of the argument and try to examine whether the increased incidence of thyroid cancer was due to enhanced detection only or also a result of environmental and lifestyle risk factors.

## 2. Factors Leading to Increased Detection of Thyroid Cancer

### 2.1. Health Care Utilization

Thyroid cancer incidence has dramatically increased after the implementation of routine screening in healthy people. In 1999, South Korea initiated a national screening program for cancer. Subsequently, the national rate of thyroid cancer diagnoses reported in 2011 was 15 times of that observed in 1993, while thyroid cancer-related mortality remained stable [11]. Putting a break on the decade of explosive growth in thyroid cancer cases, a group of South Korean physicians formed a coalition in March 2014 to call for prevention from overdiagnosis of thyroid cancer, and screening with ultrasonography in healthy people was called into question. Subsequently, insurance claims data suggested a 30% reduction in the incidence of thyroid cancer in South Korea [12].

Studies also found that thyroid cancer incidence would be elevated in communities with higher household income, education, and health insurance coverage [13]. In an analysis of the Surveillance, Epidemiology, and End Results (SEER) data, papillary thyroid cancer (PTC) incidence was correlated with sociodemographic markers of health care access. It was positively correlated with rates of college education, white-collar employment, and family income and negatively correlated with the percentage of residents who were uninsured, in poverty, unemployed, of nonwhite ethnicity, non-English speaking, and lacking high school education [14]. In another study using the 1999–2009 U.S. National Program of Cancer Registries data, the incidence rate of thyroid cancer was correlated with the population density of endocrinologists and general surgeons in the area, as well as the use of ultrasonography [15]. Approximately half of the variability in state-level thyroid cancer incidence could be explained by the three factors, though underestimation due to misclassification may have occurred as otolaryngologists were not captured in the database, and some general surgeons or endocrinologists may not focus on the thyroid.

Taken together, these studies suggest that health care access and utilization had led to increased detection and could explain some of the variability in thyroid cancer incidence.

### 2.2. Increased Use of Imaging Examination

Thyroid nodules are found in 30%–50% of the population in late adulthood [16]. Therefore, incidental thyroid nodules (including malignant nodules) are frequently identified upon physical examination or diagnostic procedures for other primary diseases. In the past 30 years or so, the advancement in imaging technology has made possible the identification of many previously undetectable conditions via the use of imaging modalities.

Individuals reportedly underwent an average of 1.18 imaging studies per year in the U.S. [17]. Of all the imaging scans, 35% were advanced diagnostic imaging studies such as computed tomography (CT), magnetic resonance imaging (MRI), ultrasound, or nuclear medicine, including positron emission tomography (PET). Over the 15 years of the study, imaging rates for CT scans increased 8% annually. During 2004 to 2010, MRI rates increased 10%, ultrasound 4%, and PET scans 57%, annually. In the U.S. study of the thyroid cancer “epidemic” and density of endocrinologists, the incidence rates were also found to be significantly correlated with the use of cervical ultrasonography in both males and females [15]. It is likely that increased use of imaging examination had led to the identification of an increasing number of otherwise clinically occult thyroid diseases.

### 2.3. Increased Frequency and Extent of Thyroid Surgery

In the U.S., the annual number of thyroidectomies increased 39%, from 66,864 cases in 1996 to 92,931 cases in 2006. Surgery (total or subtotal thyroidectomy) was more frequently performed than before for nonmalignant thyroid diseases, such as hyperthyroidism or benign thyroid nodules [18]. In a retrospective study which enrolled patients who had undergone total thyroidectomy for hyperthyroidism, incidental papillary thyroid microcarcinomas (PTMC) were found in 28% of the patients in the euthyroid goiter group and 26% of the patients with Graves' disease [19]. Increased number of thyroidectomies meant more specimens to be examined for carcinoma, possibly another contributing factor for increased detection.

An analysis based on SEER data between 1998 and 2010 suggested that an increasing number of patients underwent total thyroidectomy for PTMC, yet 5- and 10-year overall survival was similar to that of the general population, indicating overtreatment for an otherwise indolent condition [20]. The American Thyroid Association (ATA) released an updated practice guideline in 2015, suggesting active surveillance management as an alternative to immediate surgery in low-risk, asymptomatic patients with PTMC [21]. We might begin to see reductions in the number of thyroid operations and perhaps the incidence of thyroid cancer.

### 2.4. Changed Practice of Pathological Examination

Pathological examinations for thyroid specimens are now more thorough, since pathology reporting guideline increased in complexity and completeness. In the past, only certain sections of a specimen were examined by a pathologist. In contrast, the thyroid specimen in its entirety is now typically examined, increasing the chances for the identification of small cancers. Along with the increased number of thyroid surgery, more thyroid specimens were examined, which in turn may have led to increased detection [18]. Moreover, cases were increasingly classified as “follicular variant of papillary carcinoma” rather than follicular adenoma [22, 23], which may also partly explain the increased number of PTC. Acknowledging the trend of “overclassification” of thyroid nodules as cancer, the scientific community made an effort to reduce unnecessary aggressive treatment: encapsulated noninvasive follicular variants of papillary thyroid carcinoma (FVPTC) have recently been reclassified as a benign entity and renamed as “noninvasive follicular thyroid neoplasms with papillary-like nuclear features” (NIFTP) to reduce overdiagnosis and overtreatment of indolent cases [24].

## 3. Risk Factors for Thyroid Cancer

Although the trend of increased detection seemed to have growth patterns parallel to the increased incidence of thyroid cancer, evidence also supports a true increase of thyroid cancer incidence beyond overdiagnosis. U.S. studies using linear correlation analysis [14, 15] showed that approximately half of the variability in state-level incidence of thyroid cancer could not be explained by the “overdiagnosis” theory.

Moreover, even with early detection and treatment, the incidence of large tumors and cancer-related mortality also slightly increased [25], suggesting other factors leading to the growth of thyroid cancer incidence. Therefore, environmental/lifestyle factors (e.g., radiation, iodine intake, and nitrates), as well as comorbidities (e.g., chronic lymphocytic thyroiditis), and perhaps a complex multiplication of these factors are considered possible causes for a true increase in thyroid cancer incidence.

### 3.1. Radiation Exposure

After the nuclear power accident of Chernobyl in 1986, a large increase in childhood thyroid cancer incidence was reported in contaminated areas [26]. The thyroid gland may be more easily irradiated than other tissues because of its position in the body and its propensity to absorb iodine. In particular, the thyroid is characteristically radiosensitive at a young age [27, 28]. After acute exposure (e.g., energy released by an atomic bomb) before age 20, the excess relative risk of thyroid cancer has been found to persist for over 50 years [29].

Ionizing radiation causes DNA strand breaks and somatic mutations that are thought to be a risk factor for cancer in general [30]. Medical sources of thyroid-related radiation include diagnostic or therapeutic procedures, such as X-rays, CT scans, and ^131^I therapy, and the use of which has been on the rise with increasing number of imaging interventions. During the last 25 years, the radiation dose per person has doubled in the U.S. [31]. A small case-control study suggested that dental X-rays, particularly multiple exposures, were associated with increased thyroid cancer risk in Kuwaiti patients [32]. Nevertheless, the history of radiation exposure is only found in a small proportion of thyroid cancers.

On the molecular level, the evidence seems more telling. Radiation-induced PTC is unique in its molecular profile that most cases were found to have RET-PTC chromosomal rearrangements, while BRAF or RAS point mutations, commonly found in sporadic PTCs, rarely occurred [26]. Molecular studies across diverse populations have indicated a decrease in radiation-induced PTCs and an increase in sporadic PTCs [33, 34].

While radiation exposure, either from the environment (such as nuclear leakage) or medical diagnostics, may have contributed a few extra cases of thyroid cancer diagnosed every year, it could not have explained the sharp increase in thyroid cancer incidence. Furthermore, the association between medical radiation exposure and thyroid cancer risk may be confounded by factors such as individual susceptibility, iodine nutritional status, and patient selection bias. How these factors may mediate or influence the risk of radiation exposure in relation to thyroid cancer is unclear.

### 3.2. Iodine Intake

Iodine is an essential element for the synthesis of thyroid hormones. Since universal salt iodization was introduced in the 1990s, whether iodine contributes to the increasing cancer incidence has been a topic of controversy and public concern.

Animal studies have demonstrated an increase in the incidence of thyroid epithelial cell carcinomas after prolonged iodine deficiency. It was shown that Syrian hamster [35] and mice [36] on an iodine-restricted diet were more likely to develop thyroid cancer than those on a low-iodine diet. On the other hand, in vitro study showed that iodine could promote the growth of thyroid cancer cells with the increase of iodine concentration in a specific range [37].

Epidemiological studies also had conflicting results, including several case-control studies conducted in French Polynesia, California, Hawaii, and New Caledonia [38–41]. A higher dietary iodine intake was significantly associated with a decreased risk of thyroid cancer in French Polynesia [38]. In California, iodine exposure appeared to have a weak effect on the risk of PTC [39]. In Hawaii, high-iodine intake was a risk factor for thyroid cancer among women [40]. There was no significant association of iodine intake and thyroid cancer risk in New Caledonia, a pacific island nation, but a slight increase in the risk of thyroid cancer was observed among those with high consumption of fish in iodine-nondeficient areas [41].

Several Chinese studies have pointed out a temporal association between the introduction of mandatary universal salt iodization in 1996 and a subsequent increase in the incidence of PTC [42]. In a large five-year prospective study between 1999 and 2004, Teng and his team found that the region with excessive iodine intake had 13 new cases of thyroid cancer, while none were diagnosed in regions with mildly deficient iodine intake or more than adequate intake [43]. In 2011, when China has reportedly eliminated iodine deficiency disorders (IDD), another cross-sectional study [44] was performed in cities with adequate iodine intake or more than adequate intake. The prevalence of thyroid nodules was much higher (12.8% versus 2.78%) compared with the prospective study of 1999–2004. Those could potentially be cancer lesions, although the nature of the nodules was not reported. A Danish study comparing thyroid cancer incidence before and after iodine supplementation showed that the incidence began to increase before the implementation of the program and continued to increase afterwards, but the magnitude of increase in incidence was stronger in the years before the implementation [45].

Level of iodine intake affects thyroid functions, but mechanisms linking with thyroid cancer are not clear. Chronic stimulation of the thyroid-stimulating hormone (TSH) and BRAF mutations in PTC are possible pathways [46, 47]. Also, some studies suggested that iodine intake may influence the distribution of thyroid cancer subtype, rather than the overall incidence. There may be more follicular and fewer papillary carcinomas in iodine-deficient areas [39] and more papillary subtype in iodine-rich areas [48, 49].

### 3.3. Obesity and Diabetes

The trend of rising thyroid cancer incidence over the past few decades also coincides with the growing trend of obesity and diabetes [50], but whether or how they are correlated is largely unknown.

A pooled analysis of prospective cohort studies found a combined hazard ratio of 1.53 for thyroid cancer in obese men and women (BMI ≥30 kg/m^2^) [51]. A meta-analysis of 21 observational studies found obesity associated with increased PTC risk and reduced medullary thyroid cancer risk [52]. In another meta-analysis, a 5 kg/m^2^ increment in BMI was strongly associated with increased thyroid cancer risk in men (RR = 1.33) [53]. However, in case-control genomic studies, selected obesity-related genetic variants were not linked to PTC risk [54].

Diabetes is associated with increased risks of various forms of cancer, including colon cancer, pancreatic cancer, breast cancer, bladder cancer, and prostate cancer [55–58]. There is, however, no established association of diabetes with thyroid cancer risk. A meta-analysis of 14 cohort and 3 case-control studies showed that women with preexisting diabetes had an increased risk of thyroid cancer, compared with their nondiabetic counterparts [59]. However, a diagnosis of diabetes may be associated with increased screening that led to increased detection of thyroid cancer, rather than contributing to a true increase in cancer incidence [60]. Lending support to a null association, a large U.S. cohort study, with a median follow-up of 15.9 years, found no significant associations between thyroid cancer risk and diabetes (hazard ratio = 1.09; 95% confidence interval, 0.79–1.52), diabetes treatment, or duration of diabetes, among postmenopausal women [61].

Mechanisms for a possible link between obesity, diabetes, and thyroid cancer include elevated levels of insulin resistance and TSH. Insulin resistance may activate insulin and the IGF pathway, which are important to cell proliferation and apoptosis [62, 63]. The chronically elevated circulating insulin levels may influence thyroid cancer risk mediated by insulin receptors overexpressed by cancer cells. However, the specific effects of hyperinsulinemia and insulin resistance on promoting thyroid cancer risk are not well understood.

### 3.4. Estrogen and Reproductive Factors

Estrogen has historically been proposed as a potential mechanism mediating the risks of breast cancer [64], endometrial cancer [65], and ovarian cancer [66]. Given that women account for over three-fourth of the prevalence of thyroid cancer, estrogen is regarded as a possible risk factor [67]. The exposure to exogenous estrogen has also seen an increasing trend due to various medical and environmental sources, including oral contraceptives, hormone replacement therapy, and consumption of meat from animals treated with growth hormone. Evidence linking these to thyroid cancer risk is, however, nonconclusive.

Cellular studies have shown that estrogen and its receptors play an important role in the proliferation, migration, and invasion of thyroid cancer [68–70]. It exerts its growth-promoting effect via a membrane-bound estrogen receptor (ER). ER*α* activation seems to induce the development of thyroid cancer, while wild-type ER*β* (ER*β*1) plays a protective role against thyroid cancer [71]. However, such effects or pathways have not been successfully demonstrated in human studies.

One case-control study showed estrogen-DNA adducts were higher in the urine of women with thyroid cancer compared to women without. The findings indicate that estrogen metabolism is unbalanced in thyroid cancer patients and suggest that formation of estrogen-DNA adducts might play a role in the initiation of thyroid cancer [72]. A recent study showed that thyroid cancer survivors were at greater risks for developing estrogen receptor-positive breast cancer than the general population. This may reflect the influence of estrogen hormone in the development of these cancers, but no definitive relationship has been established between thyroid disorder and breast cancer [73]. However, a multicenter prospective cohort study found that women who had had hysterectomy had a significantly higher risk of thyroid cancer compared with women with no history of hysterectomy; and among women who had hysterectomy, HRT was associated with a lower risk of thyroid cancer. More research is needed to clarify whether these associations were confounded by indications for hysterectomy [74].

### 3.5. Hashimoto's Thyroiditis

The incidence of chronic autoimmune Hashimoto's thyroiditis (HT) has increased in the past two decades, paralleling the trend of increased thyroid cancer incidence [75]. Yet the link between HT and PTC has long been a topic of controversy.

Some plausible mechanisms have been proposed. Elevated levels of TSH found in hypothyroid patients with autoimmune thyroid disease may stimulate follicular epithelial proliferation, promoting the development of papillary carcinoma. Autoimmune thyroiditis might also induce thyroid tumorigenesis via the production of proinflammatory cytokines and oxidative stress [76]. Histologically, follicular epithelial dysplasia in the form of scattered microfollicles lacking of follicular colloid or irregularly shaped follicles in HT tissues was observed. There are also several similarities between HT and PTC in cytological and immunomarker profiles [77].

However, studies using fine-needle aspiration cytology (FNAC) and those using thyroidectomy specimen showed conflicting results. Studies using FNAC specimens showed no significant increase of PTC in patients with HT [78–81], whereas surgical series using thyroidectomy showed a risk for coexistent PTC [82–86]. This may possibly have resulted from the fact that, in the FNAC studies, the specimens were limited by lack of definitive histological pathology and/or from selection bias of the surgical specimens from patients with comorbidity [87].

### 3.6. Lifestyle Factors

Besides iodine intake, the changing diet and growing varieties of food additives may also be associated with the incidence of thyroid cancer. For example, nitrate, with its increasing presence in our dietary composition, was postulated to be a risk factor for thyroid cancer [88]. Dietary nitrate is found in cured meats, various types of vegetables, and as contaminant of drinking water, thereby potentially posing bigger public health risks [89, 90]. Nitrate is regarded as a plausible risk factor for thyroid cancer, as it competitively inhibits iodide uptake by the thyroid, potentially affecting thyroid functions [91]. Some epidemiologic evidence is available to demonstrate elevated thyroid cancer risk linked to excess dietary nitrate intake. A U.S. retrospective cohort study of older women in the agricultural state of Iowa found an increased risk of thyroid cancer with higher average nitrate levels in public water supplies and with longer consumption of water exceeding 5 mg/L nitrate-N [92]. Researchers of a large U.S. prospective cohort of retired persons [93], assessing dietary intake by using food frequency questionnaire, found nitrate intake associated with an increased risk of thyroid cancer among men, but not among women.

Physical activity is commonly thought to play a role in mediating cancer risk. Moreover, many chronic conditions are believed to be attributable to an increasingly sedentary lifestyle. Physical activity has been hypothesized to influence thyroid cancer risk through several mechanisms [94], for example, by improving endogenous DNA repair capacity, reducing body fat, lowering insulin resistance, and altering circulating inflammatory factors [95–97]. In two case-control studies, the risk of thyroid cancer was slightly reduced among subjects who reported recreational physical activity; whereas weekly frequency (hours/week) seemed to be more relevant than duration (years) in reducing risk [98]. However, in a meta-analysis, the summary of relative risk estimates from cohort and case-control studies combined indicated no association between physical activity and thyroid cancer risk [50].

There is some evidence that cigarette smoking may be associated with thyroid cancer risk. The results of pooled analysis of five prospective studies suggest that cigarette smoking is associated with a 30–40% reduced risk of papillary thyroid cancer and, possibly, follicular thyroid cancer [99]. Cigarette smoking could potentially influence thyroid cancer risk by altering thyroid stimulating hormone, serum thyroid antibodies, and sex steroid hormone level [100, 101]. As all the studies used self-administered questionnaires, selection and recall bias may have partly contributed to the null association.

## 4. Conclusion

Widespread screening leading to detection of otherwise clinically occult cases is believed to have contributed to an apparent increase of thyroid cancer incidence globally. However, epidemiological, biological, and clinical data do not support the apparent increase as the exclusive explanation of growth in thyroid cancer incidence and suggest that a true increase of cases has occurred.

In an effort to control and combat the increasing trend of thyroid cancer, much effort has been devoted to the search for modifiable risk factors of this malignancy. Among the wide range of studied factors hypothetically affecting thyroid cancer risk, early childhood exposure to iodine radiation is the only well-established risk factor. This could possibly be explained by the unparalleled magnitude of the exposure at a developmental stage of the thyroid glands in those studies. The analyses of other potential risk factors have not presented with conclusive evidence, and studies of the disease mechanisms often indicate multiple pathways/agents that may or may not play a significant role in a given population. For example, in addition to age of exposure, epidemiologic evidence also points to gender and geographic differences in risk estimates. Comorbidity and individual susceptibility may also differ when it comes to disease mechanisms, given the complex endocrine system. Hence, it is probably not entirely surprising to have had conflicting reports on the same variable when there was not sufficient control of the noise.

Adding to the challenge to sift through potential confounders is the imprecise measurement of behavioral factors, including dietary/nutrient intake, smoking, and level of physical activity. The limitations in measurement are almost inevitable in epidemiologic studies. However, we believe that traditional methods of self-report cannot sufficiently capture these behavioral factors, as the magnitude of potentially carcinogenic effect is likely to be washed out when multiple forces are at play. To better control for confounding and to arrive at better risk estimates, future studies may need to use objective measurements, for example, biomarkers, and focus on testing a specific agent or a specific value range of the exposure among a defined population group. Furthermore, it is now possible to reclassify thyroid cancer into molecular subtypes that better reflect the underlying signaling and differentiation properties [102]. This presents an opportunity in cancer case restratification, which may help researchers in linking modifiable risk factors to a more defined subgroup of patients.

Lastly, enhanced detection may have led to the identification of otherwise clinically irrelevant cases; but a nodule size of <1 cm does not predict indolence, and papillary thyroid microcarcinomas could also represent biologically aggressive disease [103]. It is of clinical and public health importance to be able to differentiate stationary cancers from potentially aggressive diseases.
